# Dislocation Substructures Evolution and an Informer Constitutive Model for a Ti-55511 Alloy in Two-Stages High-Temperature Forming with Variant Strain Rates in β Region

**DOI:** 10.3390/ma16093430

**Published:** 2023-04-27

**Authors:** Shen Tan, Daoguang He, Yongcheng Lin, Bingkun Zheng, Heyi Wu

**Affiliations:** 1 School of Automation, Central South University, Changsha 410083, China; shiina1024@csu.edu.cn; 2 School of Mechanical and Electrical Engineering, Central South University, Changsha 410083, China; kakigaku1024@gmail.com (B.Z.); shiina1024@163.com (H.W.); 3 State Key Laboratory of Precision Manufacturing for Extreme Service Performance, Changsha 410083, China

**Keywords:** hot deformation, informer deep learning model, microstructural evolution, titanium alloy

## Abstract

The high-temperature compression characteristics of a Ti-55511 alloy are explored through adopting two-stage high-temperature compressed experiments with step-like strain rates. The evolving features of dislocation substructures over hot, compressed parameters are revealed by transmission electron microscopy (TEM). The experiment results suggest that the dislocations annihilation through the rearrangement/interaction of dislocations is aggravated with the increase in forming temperature. Notwithstanding, the generation/interlacing of dislocations exhibit an enhanced trend with the increase in strain in the first stage of forming, or in strain rates at first/second stages of a high-temperature compressed process. According to the testing data, an Informer deep learning model is proposed for reconstructing the stress–strain behavior of the researched Ti-55511 alloy. The input series of the established Informer deep learning model are compression parameters (compressed temperature, strain, as well as strain rate), and the output series are true stresses. The optimal input batch size and sequence length are 64 and 2, respectively. Eventually, the predicted results of the proposed Informer deep learning model are more accordant with the tested true stresses compared to those of the previously established physical mechanism model, demonstrating that the Informer deep learning model enjoys an outstanding forecasted capability for precisely reconstructing the high-temperature compressed features of the Ti-55511 alloy.

## 1. Introduction

Due to its outstanding properties consisting of mechanical properties, anticorrosive performance, and thermal treatability, near-β titanium alloy is comprehensively applied in the crucial manufacture of load-bearing aircraft components [1,2]. Usually, hot deformation is necessarily utilized to improve the microstructures and further optimize the practical performance of titanium alloys [3,4,5]. The coupling effects of multiple forming parameters induce intricate evolving characteristics of microstructures and high-temperature flow behavior of titanium alloys [6,7,8,9,10]. Hence, investigations on the microstructural evolution and accurately modeling the true stress–strain characteristics of titanium alloys are significant.

To this day, numerous investigations have been devoted to exploring the microstructural evolution mechanisms of titanium alloys [11,12,13,14,15]. Some reports [16,17] revealed the substructural evolving features for multiple titanium alloys in thermal forming and detected that the substructural nucleated/migration mechanisms were substantially affected by processing parameters. Meanwhile, it was found that the evolution of substructures could exert a prominent effect on the nucleated/coarsening of dynamic recrystallization (DRX) [18,19,20,21]. Additionally, the transformation mechanisms of phases (i.e., α phase globularization [22,23], α phase conversion into β phase [24,25]) were intensively analyzed. As mentioned in previous investigations, intricate microstructural variation/interaction characteristics frequently emerge and notably affect the thermal forming features of titanium alloys.

Describing high-temperature flow characteristics of alloys is a current research subject and obtained tremendous achievements with various constitutive models [26,27,28,29,30,31,32,33]. First, multiple phenomenological models were constructed/improved for reproducing the thermal flow features of alloys [34,35,36,37,38]. Moreover, according to microstructural variations over processing parameters, multitudinous physical mechanism correlation models were constructed for reproducing the thermal flow characteristics of alloys [39,40,41,42]. Usually, the above two types of models can score decent prediction results, but it is challenging to formulate appropriate expressions and determine accurate material constants. Therefore, numerous machine learning models were established to simplify the conducting process and had overall superior forecasted results. For instance, the e-insensitive support vector regression (e-SVR) obtained decent results for forecasting flow characteristics [43,44]. Furthermore, complex artificial neural network (ANN) models were leveraged to predict the flow stress of titanium alloys, such as Ti600, Ti60, Ti40, and Ti-2Al-9.2Mo-2Fe β alloys [45,46,47]. Specifically, Ge et al. [48] leveraged the artificial neural network to propose the accurate constitutive model for the β-γ TiAl alloy. In recent years, various ANN-based deep learning models (DLMs) were developed to be applied in forecasting tasks, e.g., the recurrent neural network (RNN) [49,50] and long short-term memory (LSTM) [51,52,53]. However, the overfitting issue and long-term predicting performance degradation make them difficult to apply in practical usage. To tackle the problems, the Transformer-based Informer [54] deep learning model was proposed and showed an excellent capability in lithium-ion battery estimation [55]. Therefore, in this study, the two-stage high-temperature forming with variant strain rates in the β region of a Ti-55511 alloy is investigated. The Informer deep learning model was established for characterizing the microstructures and flow features of the Ti-55511 alloy.

Despite the comprehensive investigation of evolving characteristics of flow behaviors and microstructures for titanium alloys in thermal deformation at constant strain rates, systematic investigations of thermally compressed features of titanium alloys under variant strain rates remain lacking. Owing to the influences of sophisticated die structure as well as friction conditions between the die and component, the component commonly undergoes thermal forming with varying strain rates in the actual manufacturing process. Thereby, the stress–strain features for a Ti-55511 alloy in thermal compression with step-like strain rates were investigated. Furthermore, the evolving features of substructures are earnestly analyzed. Additionally, an Informer deep learning model is proposed for reconstructing the thermally compressed features of the Ti-55511 alloy.

## 2. Experimental Material and Procedure

**Figure 1:**
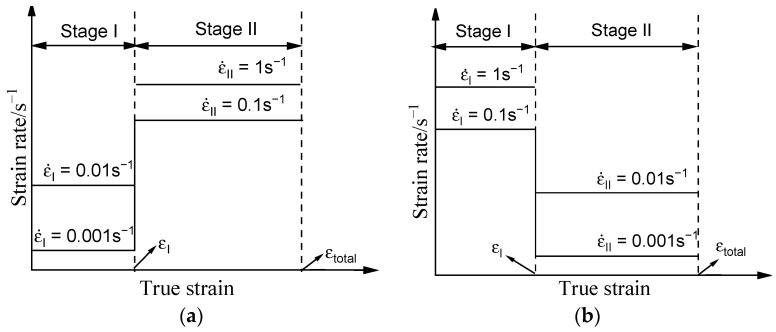
Tested steps of the received titanium alloy: (**a**) type A: the strain rates altered from the relative low values (${\dot{\varepsilon }}_{I}$) to high values (${\dot{\varepsilon }}_{\mathrm{II}}$); (**b**) type B: the strain rates altered from the relative high values (${\dot{\varepsilon }}_{I}$) to low values (${\dot{\varepsilon }}_{\mathrm{II}}$).

The commercial near-β titanium alloy was employed in the present investigation. The chemical composition (wt.%) for the researched titanium alloy was 5.16Al-4.92Mo-4.96V-1.10Cr-0.98Fe-(bal.) Ti. Cylindrical specimens (Φ8 mm × 12 mm) for thermal compression were manufactured. The Gleeble-3500 device was employed for constructing the two-stage thermally compressed experiments. Figure 1 reveals the explicit experimental procedures. Distinctly, all forming processes contain two compressed stages (I as well as II). The compressed temperature (T) and the total strain ($\varepsilon_{\mathrm{total}}$) were consistent in two stages. Here, three compressed temperatures (890 °C, 920 °C as well as 950 °C) and the constant value of $\varepsilon_{\mathrm{total}}$ (1.2) were adopted. Still, discrepant strain rates were exploited in each compressed stage. The representative complete compressed experimental step is that the specimen was thermally compressed under the strain rate of the first compression stage (${\dot{\varepsilon }}_{I}$) until the strain of stage I ($\varepsilon_{I}$) was finished, and then thermal compression was executed under the strain rate of the second compression stage (${\dot{\varepsilon }}_{\mathrm{II}}$). Correspondingly, three values of $\varepsilon_{I}$ (0.3, 0.6, as well as 0.9) were adopted.

**Figure 2:**
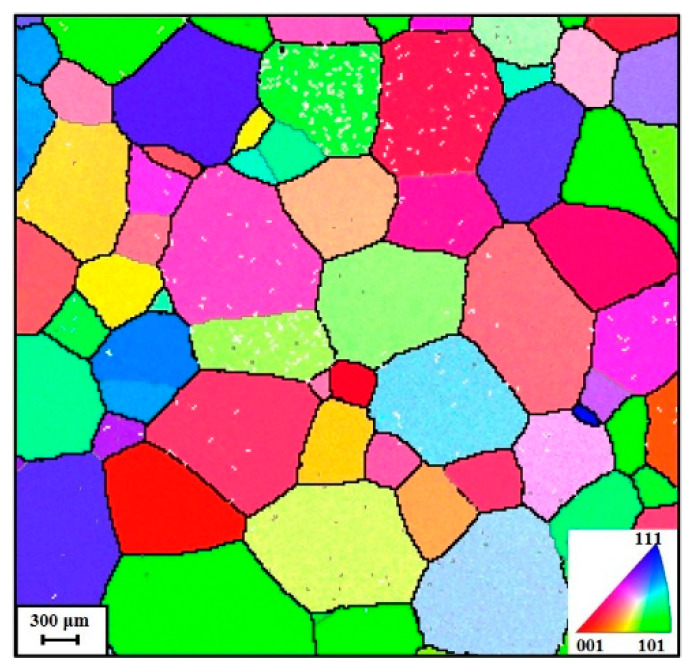
EBSD map of original grain structures in the received titanium alloy.

Before thermal compression, each sample was heated to the compressed temperatures under 10 °C/s and remained at 300 s. When the thermal compressed process was finished, the compressed blocks were directly cooled utilizing water (about 25 °C). To dissect the evolving features of substructures in thermal compression, transmission electron microscopy (TEM) was adopted. To dissect the original microstructure, electron backscatter microscopy (EBSD) was chosen. For analyzing using TEM as well as EBSD, the thermally compressed samples were axially machined for acquiring cross-sections. Afterwards, these sections were ground, polished, and etched in a solution (10 mL HClO_4_ + 70 mL C_4_H_10_O + 120 mL CH_3_OH). Figure 2 displays the original grain structures, and most of the initial β grains are equiaxed grains.

## 3. High-Temperature Compression Features and Substructural Evolution

**Figure 3:**
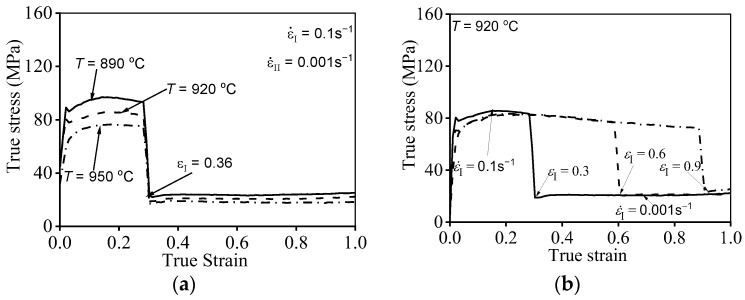
Representative flow characteristic at variation of: (**a**) T, (**b**) $\varepsilon_{I}$ [6].

**Figure 4:**
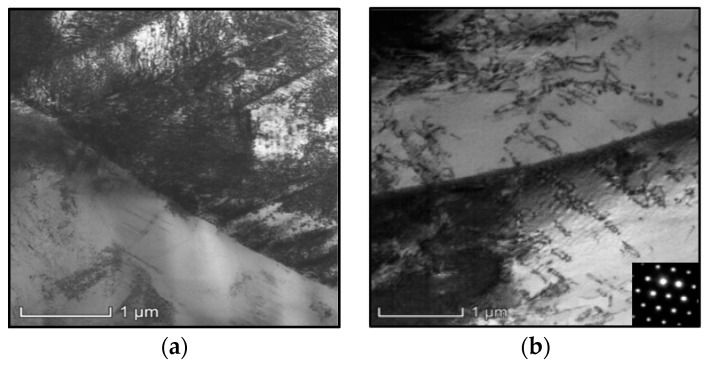

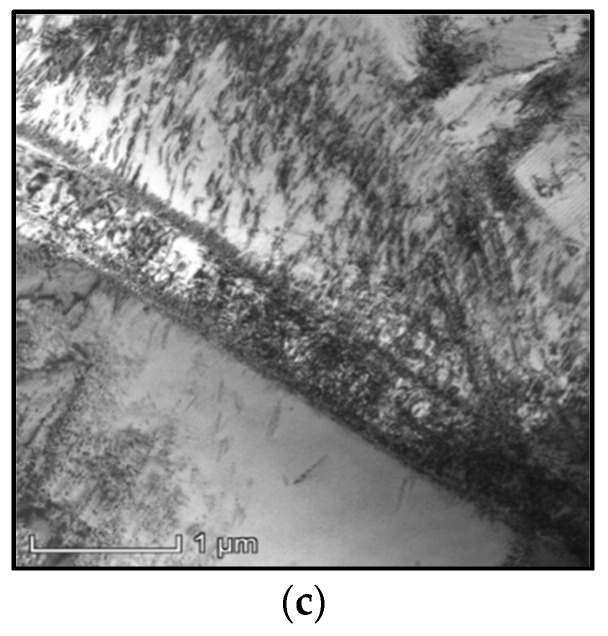
TEM figures at: (**a**) T = 920 °C/${\dot{\varepsilon }}_{I}$ = 0.1 s^−1^/$\varepsilon_{I}$ = 0.36/${\dot{\varepsilon }}_{\mathrm{II}}$ = 0.001 s^−1^, (**b**) T = 950 °C/${\dot{\varepsilon }}_{I}$ = 0.1 s^−1^/$\varepsilon_{I}$ = 0.36/${\dot{\varepsilon }}_{\mathrm{II}}$ = 0.001 s^−1^, (**c**) T = 920 °C/${\dot{\varepsilon }}_{I}$ = 0.1 s^−1^/$\varepsilon_{I}$ = 0.6/${\dot{\varepsilon }}_{\mathrm{II}}$ = 0.001 s^−1^.

The prime hot flow features of the researched titanium alloy in double-stage hot compression with stepped-strain rates are displayed in Figure 3. Clearly, the high-temperature compression behaviors are markedly affected by compression parameters. As revealed in Figure 3a, the true stresses at the first and second stages of hot compression exhibit a diminishing tendency with rising compression temperature. One principal reason for this experimental result is that the DRX behavior dramatically proceeds as the compressed temperature (*T*) ascends [6]. Moreover, the visible evolution of substructures occurs with the elevated compression temperature, as depicted in Figure 4a,b. For the compressed temperature of 920 °C and strain rate of 0.01 s^−1^, the formation of high-density dislocation clusters can be detected (Figure 4a). Then, the prominent work-hardening (WH) effect is inspired owing to the acute interaction of adjacent substructures, and the rise in true stress occurs quickly [6,16]. When the compressed temperature is elevated from 920 °C to 950 °C, the intensive migration/interaction of dislocations and grain boundary occurs, and the substructures are apparently consumed (Figure 4b). Then, the reinforced dynamic softening feature emerges with a rising incompression temperature, and a decrease in true stress appears. Furthermore, the true stress at the second stage of high-temperature compression exhibits a relative increasing trend along with the rise in the strain of the first-stage compression ($\varepsilon_{I}$), as displayed in Figure 3b. This tested result is primarily ascribed to the weakened DRX development occurring at large values of $\varepsilon_{I}$, as the strain rate is transferred from a high value (${\dot{\varepsilon }}_{I}$ = 0.1 s^−1^) to a low value (${\dot{\varepsilon }}_{\mathrm{II}}$ = 0.001 s^−1^) [6]. Meanwhile, the variations of $\varepsilon_{I}$ exerting a significant influence on the substructural evolution are depicted in Figure 4c. From Figure 4a,c, it can be detected that the generation/accumulation of substructures (subgrain, dislocation network, etc.) is promoted with increasing $\varepsilon_{I}$. Owing to the formation of high-density dislocation networks, the resistance of dislocation slippage, and grain boundary motion is raised, inducing the rise in true stress at the second stage of high-temperature compression.

## 4. The Informer Deep Learning Model for Forecasting Hot Flow Features of a Ti-55511 Alloy

In contrast to existing models with lengthy process limitations, the Transformer model demonstrates the operational potential for long sequence prediction, owing to its innovative architecture and self-attention mechanism [56]. Although the canonical self-attention mechanism is capable of processing large-scale data with impressive performance, the high computational complexity and significant memory consumption in stacking layers of the model impede its practical application. To address such a deficiency, optimized models such as the LogSparse Transformer model [57] and similar models [58] were proposed to reduce the original self-attention mechanism complexity, but their efficiency remained limited. Moreover, the Reformer model was embedded with locally sensitive hashing updated self-attention to reduce the complexity in the exceptionally long-term series for each layer [59]. In certain situations, the complexity growth rate of the Informer model was optimized to be linear, but the model could potentially experience degradation in practical long-term prediction [60]. More recently, a continuous-space attention mechanism was deployed in the Infinite Memory Transformer model to free the complexity from input length, but the prediction accuracy was decreased [61].

**Figure 5:**
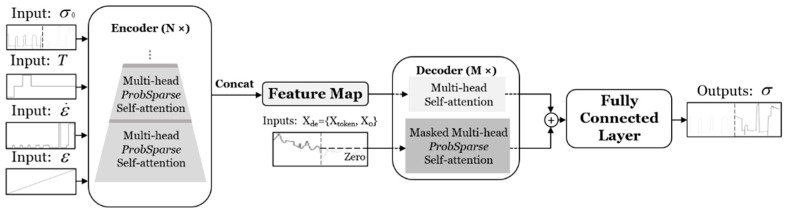
Architecture of the Informer deep learning model.

In summary, previous Transformer models focused on optimizing the complexity of the attention mechanism for each layer and obtained important findings. However, simultaneously cutting down the complexity and breaking the scalability bottleneck of stacking layers is rarely addressed. Therefore, the Informer deep learning model is proposed to address these limitations and accelerate its computing speed [54]. In the present research, the Informer deep learning model is applied as a practical method for forecasting the flow characteristics of the studied titanium alloy. Specifically, the Informer deep learning model leverages the proposed ProbSparse self-attention mechanism and distilling operation to reduce the memory usage and time complexity of the dependency alignment to $O\left(L\log L\right)$ and the space complexity to O((2−ε)LlogL). During the inference phase, the model utilizes a generative decoder form to avoid cumulative error spreading and optimize long-series output. The Informer deep learning model architecture is shown in Figure 5.

### 4.1. ProbSparse Self-Attention Mechanism

With inputs as query, key and value, the original self-attention mechanism is defined as [56],

(1) $$A\left(Q,K,V\right)=\mathrm{Softmax}\left(QK^{⊤}/\sqrt{d}\right)V$$

where $Q\in {\mathbb{R}}^{L_{Q}\times d},\;K\in {\mathbb{R}}^{L_{K}\times d},\;V\in {\mathbb{R}}^{L_{V}\times d}$, and d denotes the input dimension.

Derived by [62], the i-th query’s attention can be defined with kernel smoothing as,

(2) $$A\left(q_{i},K,V\right)=\underset{j}{\sum }\frac{k\left(q_{i},k_{j}\right)}{\sum_{l}k\left(q_{i},k_{l}\right)}v_{j}=E_{p\left(k_{j}\left|q_{i}\right.\right)}\left[v_{j}\right]$$

where $q_{i},k_{i},v_{i}$ stand for the i-th row in Q,K,V, respectively, and $k\left(q_{i},k_{j}\right)=\exp\left(q_{i}k_{j}^{⊤}/\sqrt{d}\right)$. The part $p\left(k_{j}\left|q_{i}\right.\right)=k\left(q_{i},k_{j}\right)/\sum_{l}k\left(q_{i},k_{l}\right)$ is conducted to obtain the probability, which entails a large $O\left(L_{Q}L_{K}\right)$ memory usage. Therefore, the Informer deep learning model proposed the query sparsity measurement to tackle this major defect of self-attention.

The similarity between p and q can be used to distinguish the importance, which can be conducted through Kullback–Leibler divergence as,

(3) $$KL\left(q\left\|p\right.\right)=\ln\sum_{l=1}^{L_{K}}e^{q_{i}k_{l}^{⊤}/\sqrt{d}}-\frac{1}{L_{K}}\sum_{j=1}^{L_{K}}q_{i}k_{j}^{⊤}/\sqrt{d}-\ln L_{K}$$

The measurement of the i-th query is defined by dropping the constant as,

(4) $$M\left(q_{i},K\right)=\ln\overset{L_{K}}{\underset{j=1}{\sum }}e^{\frac{q_{i}k_{j}^{⊤}}{\sqrt{d}}}-\frac{1}{L_{K}}\overset{L_{K}}{\underset{j=1}{\sum }}\frac{q_{i}k_{j}^{⊤}}{\sqrt{d}}$$

where the formula calculates the Log-Sum-Exp (LSE) and the arithmetic mean of all keys [63]. If $M\left(q_{i},K\right)$ grows larger, the probability p becomes more principal factor alterable, thus having a superior differentiating capability.

According to the above measurement, the ProbSparse self-attention mechanism can be further conducted by distributing keys to Top-*u* queries as

(5) $$A\left(Q,K,V\right)=\mathrm{Softmax}\left(\frac{\bar{Q}K^{⊤}}{\sqrt{d}}\right)V$$

where $\bar{Q}$ is the q-size sparse matrix. When $u=c\cdot \ln L_{Q}$, the layer memory usage is reduced to $O\left(L_{K}\ln L_{Q}\right)$ due to the lessened calculation for each key.

Nevertheless, the query sparsity measurement needs quadratic $O\left(L_{Q}L_{K}\right)$ calculation, and the LSE implement is not constantly numerically stable. Hence, an empirical approximation is conducted.

For each $q_{i}$, the discrete keys can be converted to continuous ones as vector $k_{j}$. In addition, the first term of the $M\left(q_{i},K\right)$ becomes the LSE of the inner product of a fixed query $q_{i}$ and all the keys, and define

(6) $$f_{i}\left(K\right)=\ln\sum_{j=1}^{L_{K}}e^{q_{i}k_{j}^{⊤}/\sqrt{d}}$$

From the Log-Sum-Exp network and relative studies [63,64], the convex function $f_{i}\left(K\right)$ combines linear $k_{j}$ for $q_{i}$, making $M\left(q_{i},K\right)$ convex. Hence, the measurement can be conducted to a derivation form with each vector $k_{j}$ as follows,

(7) $$\frac{\partial M\left(q_{i},K\right)}{\partial k_{j}}=\frac{e^{q_{i}k_{j}^{⊤}/\sqrt{d}}}{\sum_{j=1}^{L_{K}}e^{q_{i}k_{j}^{⊤}/\sqrt{d}}}\cdot \frac{q_{i}}{\sqrt{d}}-\frac{1}{L_{K}}\cdot \frac{q_{i}}{\sqrt{d}}$$

Let $\vec{\nabla }M\left(q_{i}\right)=\vec{0}$ to reach the minimum value; the condition can be listed as,

(8) $$q_{i}k_{1}^{⊤}+\ln L_{K}=\cdots =q_{i}k_{j}^{⊤}+\ln L_{K}=\cdots =\ln\sum_{j=1}^{L_{K}}e^{q_{i}k_{j}^{⊤}}$$

The minimum value $\ln L_{K}$ can be obtained when $k_{1}=k_{2}=\cdots =k_{L_{K}}$. Therefore, the measurement can be written as

(9) $$M\left(q_{i},K\right)\geq \ln L_{K}$$

Hence, by picking the largest inner-product $\max_{j}\left\{q_{i}k_{j}^{⊤}/\sqrt{d}\right\}$, the inequation can be derived as

(10) $$\left\{\begin{array}{c} M(q_{i},K)=\ln\overset{L_{K}}{\underset{j=1}{\sum }}e^{\frac{q_{i}k_{j}^{⊤}}{\sqrt{d}}}-\frac{1}{L_{K}}\overset{L_{K}}{\underset{j=1}{\sum }}\left(\frac{q_{i}k_{j}^{⊤}}{\sqrt{d}}\right)\\ \;\;\;\;\;\leq \ln\left(L_{K}\cdot \underset{j}{\max}\left\{\frac{q_{i}k_{j}^{⊤}}{\sqrt{d}}\right\}\right)-\frac{1}{L_{K}}\overset{L_{K}}{\underset{j=1}{\sum }}\left(\frac{q_{i}k_{j}^{⊤}}{\sqrt{d}}\right)\\ \;\;\;\;\;=\ln L_{K}+\underset{j}{\max}\left\{\frac{q_{i}k_{j}^{⊤}}{\sqrt{d}}\right\}-\frac{1}{L_{K}}\overset{L_{K}}{\underset{j=1}{\sum }}\left(\frac{q_{i}k_{j}^{⊤}}{\sqrt{d}}\right) \end{array}\right.$$

Eventually, by combining the above equations, the bound can be denoted as

(11) $$\ln L_{K}\leq M\left(q_{i},K\right)\leq \max_{j}\left\{q_{i}k_{j}^{⊤}/\sqrt{d}\right\}-\frac{1}{L_{K}}\sum_{j=1}^{L_{K}}\left\{q_{i}k_{j}^{⊤}/\sqrt{d}\right\}+\ln L_{K}$$

where $q_{i}\in {\mathbb{R}}^{d}$ and $k_{j}\in {\mathbb{R}}^{d}$ are in the keys set K.

From the above deductions, the max–mean measurement can be defined as

(12) $$\bar{M}\left(q_{i},K\right)=\underset{j}{\max}\left\{\frac{q_{i}k_{j}^{⊤}}{\sqrt{d}}\right\}-\frac{1}{L_{K}}\overset{L_{K}}{\underset{j=1}{\sum }}\frac{q_{i}k_{j}^{⊤}}{\sqrt{d}}$$

Specifically, a long-tail distribution pattern of the self-attention mechanism was observed by performing a qualitative assessment [54]. In this case, only a few dot product pairs contribute to the major attention. Hence, $\bar{M}\left(q_{i},K\right)$ only requires $U=L_{K}\ln L_{Q}$ dot product pairs of random sampling, and the remaining pairs are filled with zero values. Therefore, the operation has a weaker sensitivity and remains numerically stable. Eventually, in practical application, the relatively equivalent input length $L_{Q}=L_{K}=L$ in self-attention computation can reduce the complexity to $O\left(L\ln L\right)$.

### 4.2. Encoder

**Figure 6:**
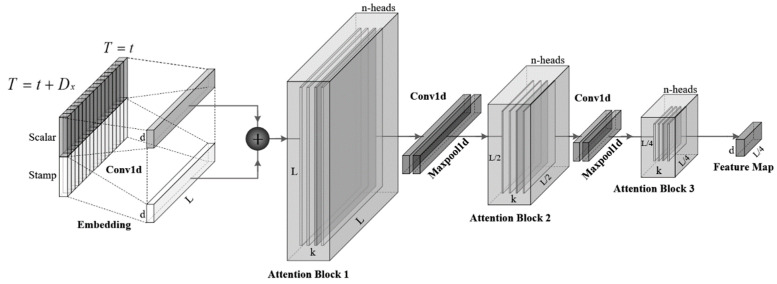
The architecture of a single stack in the encoder of the Informer deep learning model.

The Informer deep learning model utilizes the encoder architecture to extract the long-term dependency of input series, where the *t*-th input $X^{t}$ is reshaped as matrix $X_{\mathrm{en}}^{t}\in {\mathbb{R}}^{LX\times d\mathrm{model}}$ [56]. The encoder is composed of multiple identical layers stacked on top of each other. Specifically, the architecture of a single stack in the encoder of the Informer deep learning model is given in Figure 6.

Due to the processing of the ProbSparse self-attention mechanism, the encoder is loaded with redundant value V combinations. Hence, self-attention distilling is proposed to concentrate self-attention mechanisms for the next layer.

Based on the dilated convolution [65], the distilling operation feeds forwards the $\left(j+1\right)$-th layer as,

(13) $$X_{j+1}^{t}=\mathrm{MaxPool}\left(\mathrm{ELU}\left(\mathrm{Conv}1d\left({\left[X_{j}^{t}\right]}_{\mathrm{AB}}\right)\right)\right)$$

where ${\left[\cdot \right]}_{\mathrm{AB}}$ denotes the attention block, and $\mathrm{Conv}1d\left(\cdot \right)$ generates a 1D convolutional filter with $\mathrm{ELU}\left(\cdot \right)$ activation function [66].

The max-pooling layer is added to reduce the total memory usage to $O\left(\left(2-\varepsilon \right)L\log L\right)$. Furthermore, a pyramid-like processing structure (shown in Figure 6) is established where inputs are halved to serve as the replication of the main stack and the distilling layers drop gradually. In this case, the operation has a better robustness, and the resulting dimensions of different layers are consistent.

### 4.3. Decoder

The canonical decoder structure is optimized with generative inference to mitigate the long-term speed descent. The decoder mechanism is defined as

(14) $$X_{\mathrm{de}}^{t}=\mathrm{Concat}\left(X_{\mathrm{token}}^{t},X_{0}^{t}\right)\in {\mathbb{R}}^{\left(L_{\mathrm{token}}+L_{y}\right)\times d_{\mathrm{model}}}$$

where $X_{\mathrm{token}}^{t}\in {\mathbb{R}}^{L_{\mathrm{token}}\times d_{\mathrm{model}}}$ is the start token, and $X_{0}^{t}\in {\mathbb{R}}^{L_{y}\times d_{\mathrm{model}}}$ is the placeholder for target sequences.

Extended from dynamic decoding [67], the procedure is innovated to sample a $L_{\mathrm{token}}$ series in the input sequence as a start token then feed it to the decoder as $X_{\mathrm{de}}=\left\{X_{L},X_{0}\right\}$. Afterwards, the decoder obtains outputs through a single forward procedure, and thus it can process with less time consumption than a normal encoder-decoder architecture.

### 4.4. Identification for the Parameters of the Informer Deep Learning Model

The inputs of the Informer deep learning model are temperature *T* = {890, 920, 950} °C, true strain ε = {0~1}, and strain rate $\dot{\varepsilon }$ = {0.001, 0.01, 0.1, 1} s^−1^. The input sequences were preprocessed by concatenating experimental data of true stress values under different temperatures, true strains, and strain rates. The corresponding temperature, true strain, and strain rate values were also concatenated in the sequences. Then, these sequences were applied as training inputs. The experimental data are shuffled using 7/10 of the total amount for training and the rest for testing and validating the model.

As discussed above regarding the architecture of the Informer deep learning model in Section 4.1, Section 4.2 and Section 4.3 and the features of general deep neural networks, the Informer deep learning model should first be established by tuning hyper-parameters such as learning rate, input batch size, dropout, etc. To obtain the optimal parameters, the correction coefficient R, average absolute relative error AARE, mean squared error MSE, and root-mean squared error RMSE assessment criteria are employed for evaluating the results.

(15) $$R=\frac{\sum_{i=1}^{N}\left(M_{i}-\overset{•}{M}\right)\left(P_{i}-\overset{•}{P}\right)}{\sqrt{\sum_{i=1}^{N}{\left(M_{i}-\overset{•}{M}\right)}^{2}\sum_{i=1}^{N}{\left(P_{i}-\overset{•}{P}\right)}^{2}}}$$

(16) $$AARE\left(\%\right)=\frac{1}{N}\sum_{i=1}^{N}\left|\frac{M_{i}-P_{i}}{M_{i}}\right|\times 100\%$$

(17) $$MSE=\sum_{i=1}^{N}\frac{{\left(M_{i}-P_{i}\right)}^{2}}{N}$$

(18) $$RMSE=\sqrt{\sum_{i=1}^{N}\frac{{\left(M_{i}-P_{i}\right)}^{2}}{N}}$$

where N notes the total amount of result data, and $M_{i}$ and $P_{i}$ stand for the measured and predicted results when $\overset{•}{M}$ and $\overset{•}{P}$ are the mean values, respectively.

**Figure 7:**
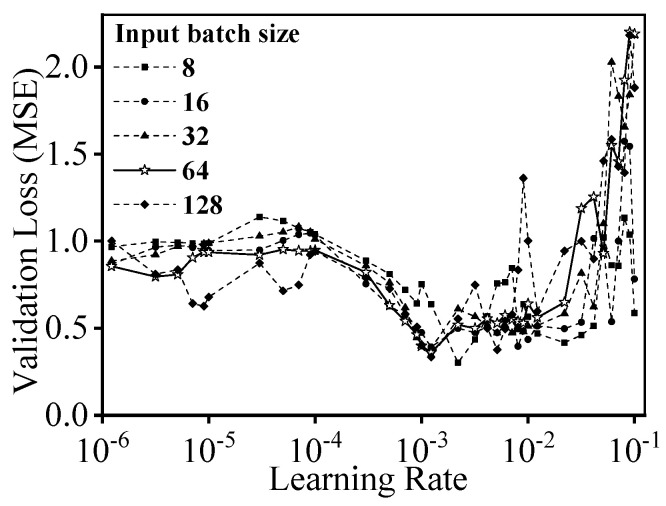
Variations of validation loss under different tested batch sizes and learning rates.

Generally, the accuracy and generalization ability of deep learning models are affected by various hyper-parameters. In the case of forecasting, the batch size of input sequences and the initial learning rate of the model play crucial roles. On the one hand, a larger batch size allows faster training but may result in worse model accuracy and an unstable training process [68]. On the other hand, a smaller batch size is beneficial for generalization but can lead to a longer computation time [69]. Additionally, both theoretical and empirical evidence have proven that the batch size and learning rate significantly impact the generalization ability and accuracy of the deep learning model [70,71,72]. To further explore the relationship between the two parameters and the results, experimental curves are displayed in Figure 7. The five curves represent the effect of the learning rate on validation loss under different batch sizes. Specifically, the learning rate is tested in a uniformly spaced range from 10^−1^ to 10^−6^ with batch sizes of 8, 16, 32, 64, and 128, respectively. The model accuracy is evaluated by the validation loss.

It is clear that the validation loss of the Informer deep learning model drops to a minimum value and then starts to fluctuate when the learning rate increases from 10^−4^ to 2 × 10^−3^. As the learning rate further ascends, the fluctuation of validation loss becomes intense, and thus the optimal learning rate can be chosen as 1.2929 × 10^−3^. Specifically, the curve fluctuations in Figure 7 demonstrate an appropriate balance of model accuracy and training stability under the batch size of 64. Hence, the batch size is determined as 64.

In addition, it is important to note that the parameters of sequence length and label length also have a significant impact on accuracy. Based on experimental results, the optimal sequence length and label length are identified as two and one, respectively.

**Figure 8:**
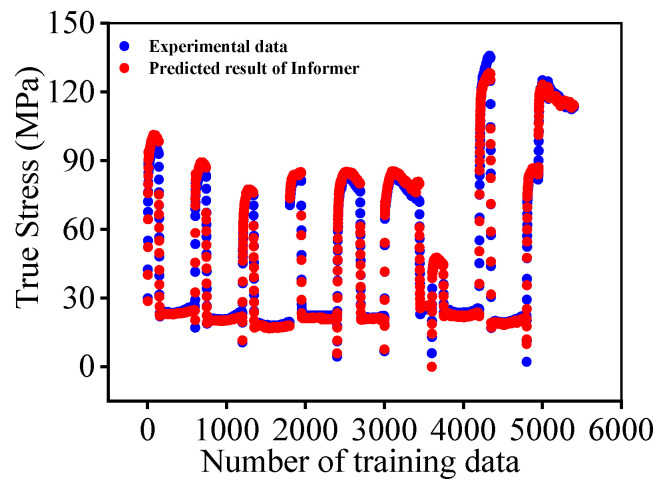
Performance of the Informer deep learning model.

Eventually, the values of *R*, *AARE*, and *RMRE* can be computed as 0.9986, 4.191%, and 2.2016, respectively. According to the results, the performance of the Informer deep learning model is shown in Figure 8. It illustrates good consistency between the experimental data and the modeled results, demonstrating the great capability of the Informer deep learning model to describe the high-temperature deformation features of the researched titanium alloy.

### 4.5. Comparisons and Discussion

As shown in the above sections, the Informer deep learning model exhibits a strong forecasting ability for the true stress of the researched titanium alloy. According to the author’s previous investigation [6], a physical mechanism (PM) model was constructed for forecasting the true stress of the researched titanium alloy, i.e.,

(19) $$\left\{\begin{array}{l} \sigma =\sigma_{y}+\sigma_{\rho }\\ \sigma_{y}=1.589{\left(\dot{\varepsilon }\exp(\frac{205,800}{RT})\right)}^{0.2052}\\ \sigma_{\rho }=4.38\times 10^{-10}\left(21.8847-0.0153T\right)\sqrt{\rho_{i}}\\ {\dot{\rho }}_{i}={\dot{\rho }}_{i}{}^{+}-{\dot{\rho }}_{i}{}^{\mathrm{DRV}}-{\dot{\rho }}_{i}{}^{\mathrm{DRX}}\\ {\dot{\rho }}_{i}{}^{+}=\frac{1}{2.86\times 10^{-10}\Lambda }\dot{\varepsilon }\\ \frac{1}{\Lambda }=\frac{1}{s}+\frac{1}{d_{i}}\\ s=\frac{F_{s}}{\sqrt{\rho_{i}}}\\ F_{s}=\begin{array}{c} 4.1797 \end{array}{\left(\dot{\varepsilon }\exp(\frac{-6.4278}{RT})\right)}^{-0.2843}\\ {\dot{d}}_{g}=2.3866d^{\begin{array}{c} 0.4468 \end{array}}\\ {\dot{d}}_{x}=-0.7803d^{0.0072}{\dot{X}}^{0.9906}\\ {\dot{d}}_{i}={\dot{d}}_{x}+{\dot{d}}_{g}\\ {\dot{\rho }}_{i}{}^{\mathrm{DRV}}=47.0321{\left(\dot{\varepsilon }\exp(\frac{0.04718}{RT})\right)}^{\begin{array}{c} -0.1259 \end{array}}\rho \\ {\dot{\rho }}_{i}{}^{\mathrm{DRX}}=\frac{0.7313{\left(\dot{\varepsilon }\exp(\frac{-9.1687}{RT})\right)}^{0.0137}\dot{X}\rho_{i}}{{\left(1-X\right)}^{2.0210}}\\ \dot{X}=\frac{6.0877M_{b}P{\left[X(1-X)\right]}^{-1.4294}{\dot{\varepsilon }}^{-2.6513}}{d^{0.5733}}\\ M_{b}=\frac{1.54\times 10^{-26}}{kT}{\left[\dot{\varepsilon }\exp(\frac{-0.1418}{RT})\right]}^{0.0045}\\ P=\frac{8.18\times 10^{-20}\rho_{i}\left(21.8847-0.0153T\right)}{2} \end{array}\right.$$

where σ is the flow stress, $\sigma_{y}$ is the short-range component, and $\sigma_{\rho }$ is the dislocation interaction stress. $\dot{\varepsilon }$ is the strain rate, R is the gas constant, *T* is the absolute temperature, $\rho_{i}$ is the dislocation density, ${\dot{\rho }}_{i}{}^{+}$ is the dislocation density emergence rate under WH, and ${\dot{\rho }}_{i}{}^{\mathrm{DRV}}$ and ${\dot{\rho }}_{i}{}^{\mathrm{DRX}}$ are dislocation density variation rate of DRV and DRX, respectively. Λ is the mean-free path of dislocation, and $d_{i}$ is the average grain size. X is the DRX fraction and the rate $\dot{X}$. $M_{b}$ is the grain boundary movement rate. P is the driving force. $D_{\mathrm{ob}}$ is the factor of self-diffusion, and δ is the grain boundary thickness.

**Figure 9:**
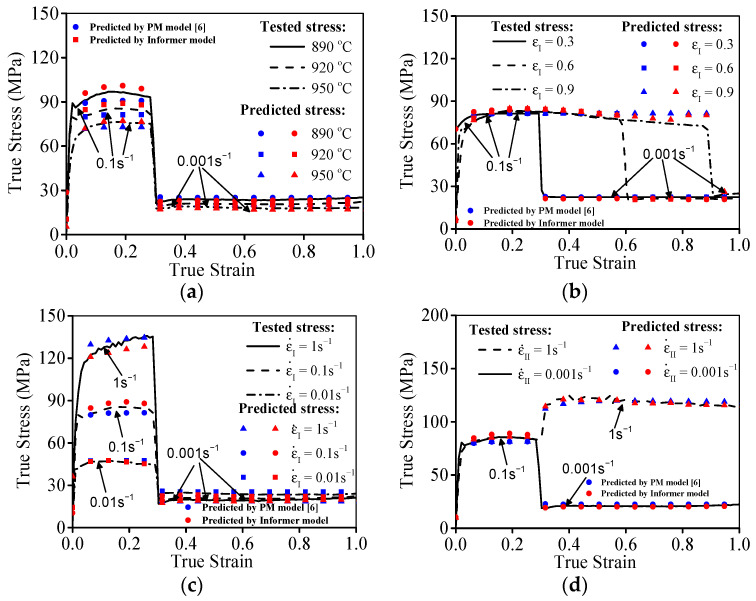
Comparisons of tested true stress and predicted results at: (**a**) *T*, (**b**) $\varepsilon_{I}$, (**c**) ${\dot{\varepsilon }}_{I}$, (**d**) ${\dot{\varepsilon }}_{\mathrm{II}}$.

**Figure 10:**
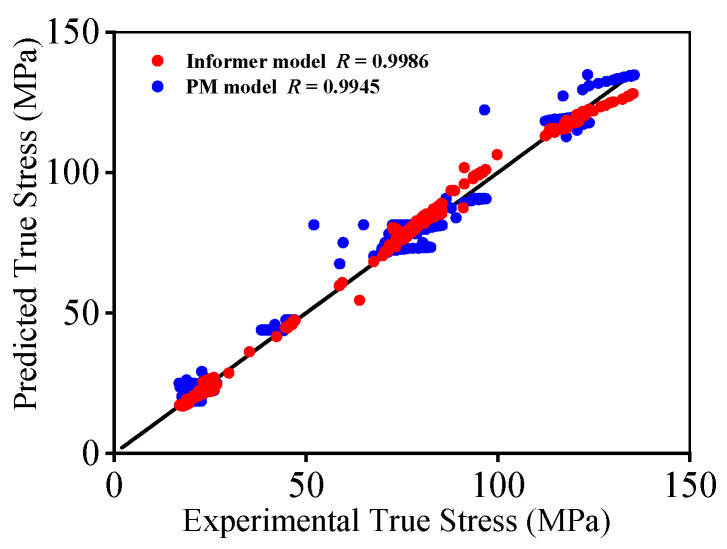
Correlation of tested true stresses and predicted values.

Figure 9 unveils the comparative analysis of forecasting performances between the PM and Informer deep learning model. Compared to that of the PM model, the Informer deep learning model enjoys a smaller forecasting error of true stresses, particularly for the researched titanium alloy at lower compressed temperature (890 °C) or higher ${\dot{\varepsilon }}_{I}$/${\dot{\varepsilon }}_{\mathrm{II}}$. To validate the forecasting capability, the correlation results of forecasted true stresses and tested ones are plotted in Figure 10. Clearly, the scatters of the PM constitutive model are more dispersed, while those of the Informer deep learning model are more centralized. Meanwhile, the values of *R*, *AARE,* and *RMSE* are determined, as noted in Table 1. Distinctly, the relative larger *R* as well as the smaller *AARE* and *RMSE* values imply that the established Informer deep learning model can accurately depict the hot compressed features of the Ti-55511 alloy.

## 5. Conclusions

The evolving characteristics of microstructures as well as flow behavior for a Ti-55511 alloy in two-stage thermal compression experiments with step-like strain rates are researched. The decisive conclusions are drawn as:

(1) In high-temperature compression, the influences of forming parameters on the flow behaviors of the researched Ti-55511 alloy are significant. Flow stresses are reduced with the increase in compressed temperature. Notwithstanding, flow stresses at stage II of thermal compression display an increase trend with the descent of $\varepsilon_{I}$ or increase in ${\dot{\varepsilon }}_{I}$/${\dot{\varepsilon }}_{\mathrm{II}}$;
(2) The formation of high-density networks/clusters through dislocation concentration/interaction is suppressed with the increase in compressed temperature. Nevertheless, the dislocation nucleation/concentration is enhanced with the increase in $\varepsilon_{I}$;
(3) The Informer deep learning model is developed to reconstruct the thermal compressed characteristics of the researched Ti-55511 alloy. The considerable agreement between the predicted true stresses and experimental results demonstrates the high prediction accuracy of the Informer deep learning model.

## Figures and Tables

**Table 1:** Calculated assessment values of the Informer deep learning model and PM constitutive model.

Model	*R*	*AARE*(%)	*RMSE*
PM model [6]	0.9945	6.181%	3.7448
Informer deep learning model	0.9986	4.191%	2.0615

## Data Availability

The raw/processed data required to reproduce these findings cannot be shared at this time as the data also forms part of an ongoing study.
